# Prognostic significance of epidermal growth factor receptor (EGFR) over expression in urothelial carcinoma of urinary bladder

**DOI:** 10.1186/s12894-018-0373-0

**Published:** 2018-06-07

**Authors:** Atif Ali hashmi, Zubaida Fida Hussain, Muhammad Irfan, Erum Yousuf Khan, Naveen Faridi, Hanna Naqvi, Amir Khan, Muhammad Muzzammil Edhi

**Affiliations:** 1 Liaquat National Hospital and Medical College, Karachi, Pakistan; 2 Brown University, Providence, RI USA; 3 Kandahar University, Kandahar, Afghanistan

**Keywords:** Bladder cancer, Muscle invasion, Epidermal growth factor receptor, EGFR, Urothelial carcinoma

## Abstract

**Background:**

Epidermal growth factor receptor (EGFR) has been shown to have abnormal expression in many human cancers and is considered as a marker of poor prognosis. Frequency of over expression in bladder cancer has not been studied in our population; therefore we aimed to evaluate the frequency and prognostic significance of EGFR immunohistochemical expression in locoregional population.

**Methods:**

We performed EGFR immunohistochemistry on 126 cases of bladder cancer and association of EGFR expression with tumor grade, lamina propria invasion, deep muscle invasion and recurrence of disease was evaluated.

**Results:**

High EGFR expression was noted in 26.2% (33 cases), 15.1% (19 cases) and 58.7% (74 cases) revealed low and no EGFR expression respectively. Significant association of EGFR expression was noted with tumor grade, lamina propria invasion, deep muscle invasion and recurrence status while no significant association was seen with age, gender and overall survival. Kaplan- Meier curves revealed significant association of EGFR expression with recurrence while no significant association was seen with overall survival.

**Conclusion:**

Significant association of EGFR overexpression with tumor grade, muscularis propria invasion and recurrence signifies its prognostic value; therefore EGFR can be used as a prognostic biomarker in Urothelial bladder carcinoma.

## Background

Bladder cancer is the fifth commonest malignancy in males all over the world [1]. Most of the bladder tumors have transitional cell (urothelial) morphology and they have a natural propensity to progress from superficial non-invasive tumors to deep muscle invasive cancers [2] . Prognosis of bladder cancer depends upon grade and stage of the disease. The single most important factor for determining disease prognosis in bladder cancer is muscle invasion; the presence of which makes therapeutic approach more radical. The only therapeutic option for muscle invasive bladder cancer is radical cystectomy or radical radiotherapy; however 5 year survival remains poor [3, 4]. On the other hand, non-muscle invasive tumors can recur and progress with time to muscle invasive disease [5]. Therefore detection of abnormal expression of biological markers is under intense scrutiny in bladder cancer, which can serve as prognostic or predictive factors in bladder cancers. Epidermal growth factor receptor (EGFR) has been shown to have abnormal expression in many human cancers and is considered as a marker of poor prognosis [6]. Frequency of over expression in bladder cancer has not been studied in our population; therefore we aimed to evaluate the frequency and prognostic significance of EGFR immunohistochemical expression in locoregional population.

## Methods

### Selection of cases

Total 126 diagnosed cases of urothelial carcinoma specimens of urinary bladder were selected from records of pathology department. Cases of bladder cancer which were diagnosed other than urothelial carcinomas were excluded. All patients underwent surgeries at Liaquat National hospital, Karachi from January 2010 till December 2014 over a period of 5 years. The study was approved by research and ethical review committee of Liaquat National Hospital and informed written consent was taken from all patients at the time of surgery. Hematoxylin and eosin stained slides and paraffin blocks of all cases were retrieved and new sections were cut where necessary. Slides of all cases were reviewed by two senior histopathologists and pathologic characteristics like tumor grade, lamina propria invasion, muscularis propria invasion were evaluated. Deep muscle invasion was analyzed when thick muscle bundles of muscularis propria were present in transurethral resection specimen. Clinical records of 58 patients were available and are thus reviewed from institutional records to evaluate recurrence and survival status. Moreover, representative tissue blocks of each case were selected for EGFR immunohistochemistry.

### Immunohistochemistry

**Fig. 1:**
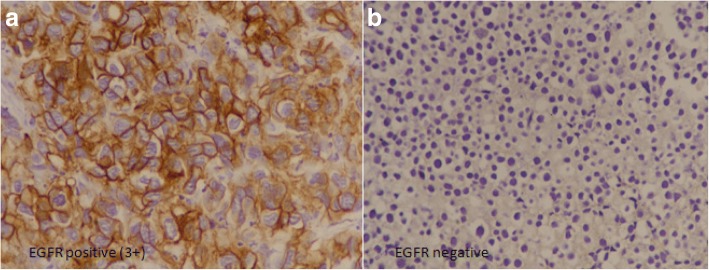
Positive (Strong & diffuse) and negative EGFR expression in bladder cancer (400× magnification)

EGFR immunohistochemistry was performed using using DAKO Monoclonal Mouse Anti-human Epidermal growth factor Receptor (EGFR), clone H11. 1:100 dilution was done using phosphate buffer saline (incubation time 30 mins at room temperature). To reduce background, incubation was done at room temperature for 20 min using proteinase K solution. Detection method was DAKO EnVision method, DAB was used as chromogen substrate (incubation time 1–3 min at room temperature) and counter stain was hematoxylin. Skin tissue was used as control. Both membranous and cytoplasmic staining for EGFR was both quantitatively and qualitatively evaluated. Intensity of staining was categorized into no staining (0), weak (1+), intermediate (2+), strong (3+) while percentage of positively stained cells were measured as continuous variable. The following scoring approach in the assessment of EGFR immunostaining was used: score 0 = no staining, unspecific staining of tumor cells or less than 10% staining, score 1 = membranous weak and incomplete staining of more than 10% of tumor cells, score 2 = moderate and complete membranous staining of more than 10% of tumor cells, score 3 = strong and complete membranous staining of more than 10% of tumor cells. Scored1+ tumors were classified as Low EGFR expression and those scored 2+ or 3+ were classified as High EGFR expression^18^ (Fig. 1).

### Follow-up and recurrence

Recurrence status and follow-up were evaluated by reviewing hospital medical records. Overall survival was taken as time from surgical excision till death or last follow-up and disease free survival was defined as time between surgical excision and local recurrence or distant metastasis, death or last follow-up.

### Statistical analysis

Statistical package for social sciences (SPSS 21) was used for data compilation and analysis. Mean and standard deviation were calculated for quantitative variables. Frequency and percentage were calculated for qualitative variables. Chi-square was applied to determine association. Student t test or Mann witney test were applied to compare difference in means among groups. *P*-value ≤0.05 as significant. Survival curves were plotted using Kaplan- Meier method and the significance of difference between survival curves were determined using log-rank ratio. *P*-value ≤0.05 was taken as significant.

## Results

Mean age of patients was 63.61 ± 14.49 years with male to female ratio of 2.6:1. 96% specimens were of transurethral resections. 48.4% (61 cases) were of high grade morphology, whereas 51.6% (65 cases) showed low grade histology. Lamina propria invasion was seen in 27.8% (35 cases), while muscularis propria invasion was noted in 17.5% (22 cases). Mean follow up of patients involved in the study was 22.76 ± 13.66 months and recurrence was seen in 43.1% (25 cases) as presented in Table 1. However, in 45 cases (35.7%) deep muscle was not present in the specimen and therefore deep muscle invasion couldn’t be assessed.

**Table 1:** Demographic profile of patients involved in the study

	n (%)
Age (years)^a^	63.61 ± 14.49
Follow up (months)^a, b^	22.76 ± 13.66
EFGR (%)^a^	14.50 ± 22.41
Gender	
Male	91(72.2)
Female	35(27.8)
Specimen Type	
Transurethral resection	121(96)
Radical Cystectomy	5(4)
Tumor Grade	
Low Grade Papillary Urothelial Carcinoma	65(51.6)
High Grade Papillary Urothelial Carcinoma	61(48.4)
Lamina Propria Invasion	
Present	35(27.8)
Absent	91(72.2)
Deep Muscle Invasion	
Present	22(17.5)
Absent	59(46.8)
Can’t Assessed	45(35.7)
Recurrence (*n* = 58)	
Yes	25(43.1)
No	33(56.9)
Survival Status (*n* = 58)	
Alive	49(84.5)
Expired	9(15.5)

^a^Mean ± SD

^b^58 cases

According to scoring system used; high EGFR expression was noted in 26.2% (33 cases), 15.1% (19 cases) and 58.7% (74 cases) revealed low and no EGFR expression respectively.

**Fig. 2:**
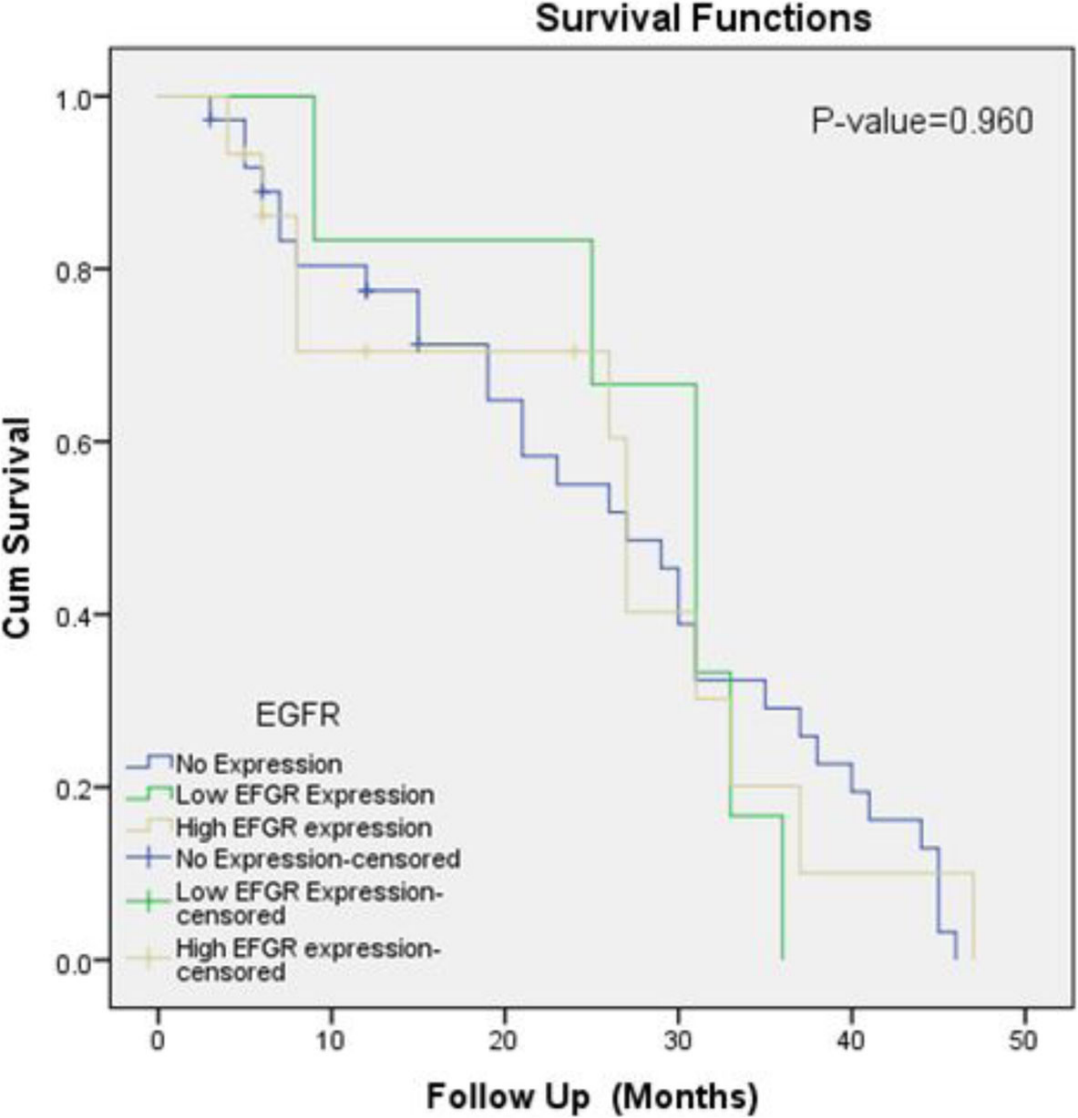
Kalpien-Meier for EGFR overexpression (overall survival)

**Fig. 3:**
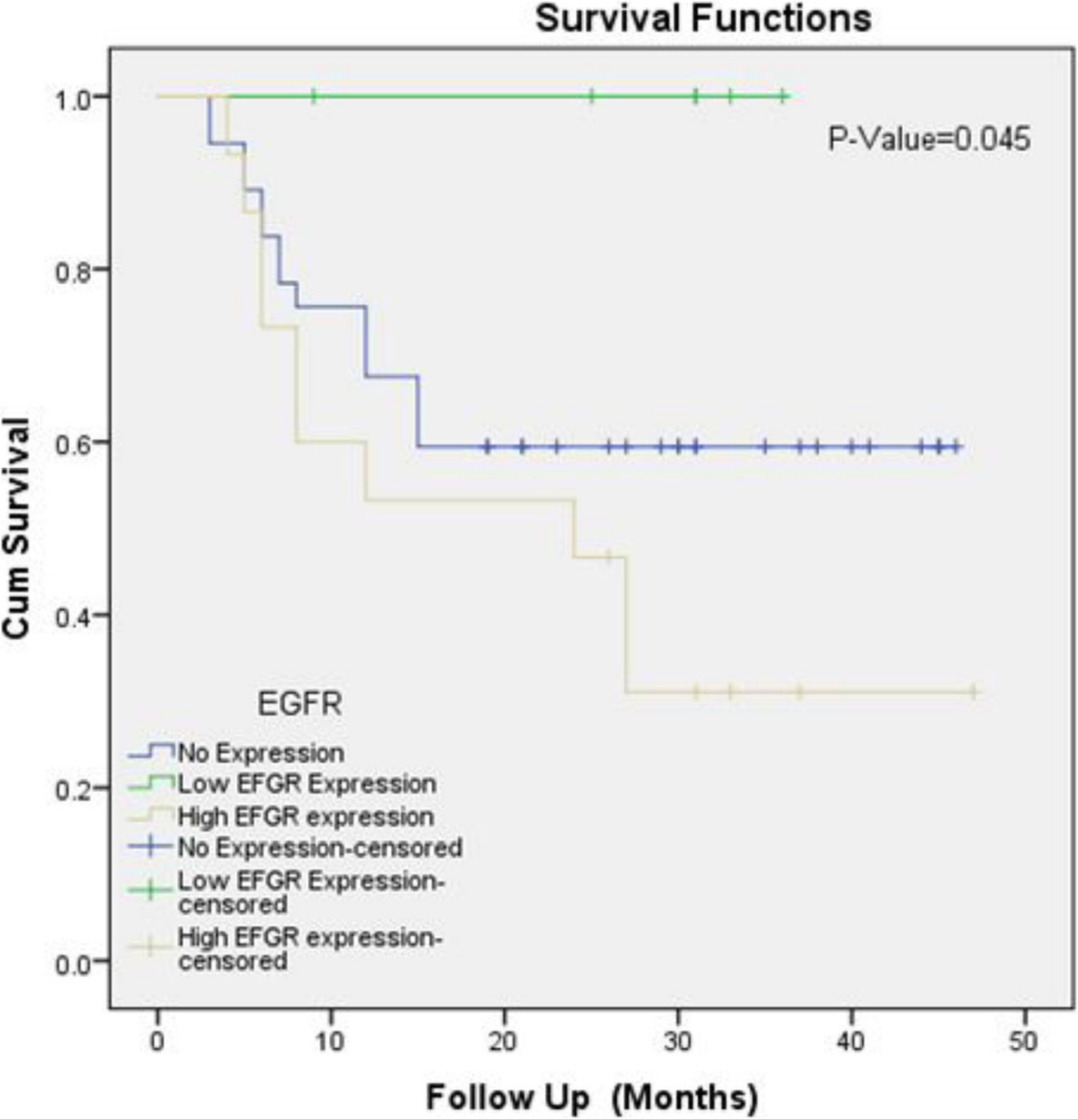
Kalpien-Meier for EGFR overexpression (recurrence)

Significant association of EGFR expression was noted with tumor grade, lamina propria invasion, deep muscle invasion and recurrence status while no significant association was seen with age, gender and overall survival (Table 2). Kaplan- Meier curves revealed significant association of EGFR expression with recurrence while no significant association was seen with overall survival (Figs. 2 and 3).

**Table 2:** Association of EGFR Expression with clinicopathologic features of Urothelial carcinoma

	EFGR Expression *n* (%)				*P*-Value
	No Expression (*n* = 74)	Low EFGR Expression (*n* = 19)	High EFGR expression (*n* = 33)	Total (*n* = 126)	
Gender					
Male	54(73)	14(73.7)	23(69.7)	91(72.2)	0.930
Female	20(27)	5(26.3)	10(30.3)	35(27.8)	
Age Group^a^					
≤ 25 years	1(1.4)	0(0)	0(0)	0(0)	0.263
26–50 years	21(28.4)	2(10.5)	2(10.5)	5(15.2)	
> 50 years	52(70.3)	17(89.5)	17(89.5)	28(84.8)	
Specimen Type ^a^					
Transurethral resection	72(97.3)	19(100)	30(90.9)	121(96)	0.209
Radical Cystectomy	2(2.7)	0(0)	3(9.1)	5(4)	
Tumor Grade					
Low Grade	48(64.9)	9(47.4)	8(24.2)	65(51.6)	0.000
High Grade	26(35.1)	10(52.6)	25(75.8)	61(48.4)	
Lamina Propria Invasion					
Present	15(20.3)	3(15.8)	17(51.5)	35(27.8)	0.002
Absent	59(79.7)	16(84.2)	16(48.5)	91(72.2)	
Deep Muscle Invasion ^a^					
Present	9(12.2)	1(5.3)	12(36.4)	22(17.5)	0.012
Absent	38(51.4)	12(63.2)	9(27.3)	59(46.8)	
Can’t Assessed	27(36.5)	6(31.6)	12(36.4)	45(35.7)	
Recurrence (*n* = 58) ^a^					
Yes	15(40.5)	0(0)	10(66.7)	25(43.1)	0.017
No	22(59.5)	6(100)	5(33.3)	33(56.9)	
Survival Status (*n* = 58) ^a^					
Alive	32(86.5)	6(100)	11(73.3)	49(84.5)	0.398
Expired	5(13.5)	0(0)	4(26.7)	9(15.5)	

Chi-Square test applied

^a^Fisher Exact test applied

*P*-Value≤0.05, considerd as significant

## Discussion

In the current study, we evaluated EGFR expression in bladder cancers of locoregional population and found EGFR expression in 46% cases of bladder cancer. Moreover, we found a significant association of EGFR expression with prognostic parameters like grade, lamina propria, deep muscle invasion and recurrence.

EGFR belongs to tyrosine kinase receptor family, all of which are encoded by c-erbB oncogenes. EGFR is the product of c-erbB1 proto-oncogene, which serves as a receptor for several growth factors like epidermal growth factor, transforming growth factor alpha, amphiregulin, heparin binding EGF like factor, betacellulin and epiregulin [7]. Activation of EGFR by one of its ligands leads to intracellular cascade of events resulting in transcriptional activation and cell proliferation [8, 9]. EGFR over expression occurs in many epithelial and solid malignancies like lung, breast and colon cancer. Several studies reported over expression of EGFR in bladder cancer. Results of most of the literature revealed that more than half of cases of bladder cancer overexpresses EGFR [10, 11]. Our data is in accordance with the reported literature; as we found significant association of EGFR expression with tumor grade, lamina propria and muscularis propria invasion; which are among most important prognostic factors in bladder cancer. Moreover, high frequency of recurrence was noted in patients with tumor showing intermediate and strong EGFR expression. Literature review revealed association of EGFR expression with high tumor grade, stage, tumor progression and poor clinical outcome [6, 12, 13]. Carisson J et al., reported 71% EGFR expression in primary bladder tumors and co-expression of EGFR and Her2neu (c-erbB2) in more than half of patients [14]. Badaway AA et al., found EGFR expression in 86% cases of bladder cancer. They reported significant association of EGFR expression with grade and tumor stage [15]. Similarly, Arfaoni AT et al., reported statistically significant correlation of EGFR expression with tumor grade and stage [16].The mechanism of EGFR over expression resulting poor prognosis is still unclear. However, evidence suggests that activation of EGFR leads to activation of activator protein of transcription factor which results in induction of matrix metalloproteinases activity [17].

## Conclusion

Significant association of EGFR overexpression with tumor grade, muscularis propria invasion and recurrence signifies its prognostic value; therefore EGFR can be used as a prognostic biomarker in Urothelial bladder carcinoma.

## Abbreviation

EGFR: Epidermal growth factor receptor
